# Optimization and Application of Electromagnetic Ultrasonic Transducer for Battery Non-Destructive Testing

**DOI:** 10.3390/s25196003

**Published:** 2025-09-29

**Authors:** Xuhang Zhan, Zhangwan Li, Hongchao Chen, Guanlin Yu, Xiaoyu Li

**Affiliations:** Key Laboratory of Optoelectronic Devices and Systems of Ministry of Education and Guangdong Province, College of Physics and Optoelectronic Engineering, Shenzhen University, Shenzhen 518060, China; 2300453018@email.szu.edu.cn (X.Z.); 2400231024@mails.szu.edu.cn (Z.L.); 2022271329@email.szu.edu.cn (H.C.); 2022271320@email.szu.edu.cn (G.Y.)

**Keywords:** lithium-ion battery (LIB), electromagnetic acoustic transducer (EMAT), non-destructive, Halbach array, design and optimization

## Abstract

The monitoring of safety and health in lithium-ion batteries (LIBs) presents a significant challenge. Ultrasonic detection techniques fulfil the requirements for high sensitivity and non-destructive evaluation in the safety assessment of these batteries. This study concentrates on the application of electromagnetic acoustic transducer (EMAT) technology for non-destructive battery testing, utilizing non-contact electromagnetic coupling to generate and receive ultrasonic waves. This method addresses the limitations associated with conventional piezoelectric ultrasonic coupling media, thereby facilitating highly reliable assessment of the internal condition of batteries. Specifically, this paper independently designs an EMAT featuring a Halbach magnet array and a butterfly coil. Based on this design, optimization is performed, and the amplitude of the received signal is increased fourfold compared to the pre-optimization configuration. The optimized transducer is employed to evaluate a set of retired batteries with a nominal capacity of 270 Ah. Experimental results demonstrate that batteries exhibiting capacities below 240 Ah produced average signal amplitudes more than 40% lower than those of batteries with higher capacities. This technology provides a non-contact, disassembly-free approach for rapid performance evaluation of batteries and demonstrates potential for effective application in sorting retired battery units.

## 1. Introduction

Lithium-ion batteries (LIBs) serve as the primary power sources in new energy vehicles, energy storage systems, and consumer electronics, owing to their superior performance, high energy density, and extended operational lifespan [1,2]. The internal health status of LIBs undergoes a complex progression throughout their entire lifecycle, characterized by several phenomena. At the electrochemical level, these include lithium plating, loss of active material (LAM) [3], and the thickening of the solid electrolyte interphase (SEI) layer [4]. Simultaneously, structural-level degradations such as electrode material cracking [5], separator aging, and electrode deformation [6] have been observed. Collectively, these degradation mechanisms contribute to reductions in battery capacity, increases in internal resistance, and an elevated risk of thermal runaway [7,8].

In the domain of retired battery assessment, there exists a critical demand for technologies capable of accurately, efficiently, and non-destructively evaluating the internal structural integrity and state of health (SOH) of batteries. The development of novel non-destructive testing methods that can comprehensively reveal key physical state information within the battery is imperative. In recent years, ultrasonic testing technology based on acoustic wave propagation characteristics has received extensive attention. This approach enables quantitative analysis and dynamic monitoring of microstructure and defect distribution within batteries. Specifically, it involves emitting elastic waves and analyzing their propagation behaviors (e.g., reflection and transmission signals) in the battery medium. A principal advantage of ultrasonic testing lies in its non-destructive nature. Thus, it allows for in-depth characterization of internal conditions without compromising the battery’s physical structure or functional performance.

Piezoelectric ultrasonic transducer (PUT), a core component of traditional ultrasonic testing, generates ultrasonic waves through the piezoelectric effect. Several scholars have applied piezoelectric ultrasonic technology to battery inspection. For example, Yi et al. [9] detected internal metal defects in LIBs by ultrasonic tomography technology. The results found that copper-based materials produce characteristic acoustic shadow artifacts and acoustic velocity shifts, enabling micron-level defect localization. This method provided a new technical approach for non-destructive quality control in battery manufacturing processes. Shen et al. [10] developed an ultrasonic scanning system that penetrates battery internal structure with high-frequency sound waves, enabling in situ monitoring and imaging of overcharging behaviors. The results revealed the correlation between the ultrasonic signal and the physical properties of the electrode, as well as side reactions such as gas evolution. This study verified the high-precision and real-time capabilities of ultrasonic technology in state of charge (SOC) monitoring and overcharge warning. Wei et al. [11] combined machine learning with ultrasonic technology and proposed an SOC estimation method for LIBs based on the initial rise time (IRT) of ultrasonic signals. The experimental results demonstrated that IRT exhibits a strong linear correlation with SOC during the discharge phase, whereas the charging phase follows a power-law relationship. This technique offered the advantage of single-channel detection compared to traditional methods, providing a novel approach for battery condition monitoring. However, traditional ultrasonic testing requires contact with the battery surface using coupling agents [12], resulting in complex operation. This method is difficult to adapt to high-temperature and high-speed inspection scenarios, thereby limiting its large-scale industrial application.

Some scholars have developed an air-coupled ultrasonic transducer (ACUT) for ultrasonic non-destructive testing. This device uses air as the coupling medium to detect surface defects or deformations by propagating ultrasound through air, thereby avoiding defects caused by traditional coupling agents. Zhang et al. [13] proposed an ACUT-based through-transmission method to defect detection in polymer LIBs. Experiments revealed that gas bubbles can reduce the transmitted wave amplitude by more than half, with a detection error of less than 5.5%. This method employed C-scan imaging to locate invisible bubbles, providing an efficient means for non-destructive quality inspection of batteries. Cho et al. [14] developed an ACUT method based on Lamb wave propagation to detect sealing defects between the lug of an LIB and the aluminum-plastic film by optimizing the critical incident angle. Their experiments verified the method’s ability to identify 1 mm foreign body defects, addressed the signal attenuation caused by air-solid impedance mismatch, and provided a new idea for packaging inspection in dry environment. Farinas et al. [15] employed non-contact ultrasonic spectroscopy technology based on high-sensitivity ACUT to simultaneously monitor SOC and mechanical integrity of batteries. Their results showed that abnormal expansion, such as gas evolution, causes the signal to disappear completely, which can serve as an early warning indicator of failure. This technology enables real-time, non-contact evaluation of dynamic battery parameters, facilitating manufacturing optimization and health management.

Other scholars use laser Doppler vibration (LDV) method to capture signals, which measures the vibration velocity or displacement of an object’s surface by analyzing changes in the frequency of reflected ultrasonic waves. This technique is also non-contact. Galos et al. [16] analyzed the influence of the embedded battery position on the modal frequency, damping ratio, and acoustic performance of composite materials through LDV experiments and finite element simulations. Their results demonstrated that embedding batteries at vibration nodes can enhance the flexural damping ratio of composite materials and increase the coincidence frequency, thereby optimizing acoustic behavior. This research provides both experimental and theoretical support for the lightweight design of electric vehicles. Zheng et al. [17] proposed an in situ imaging technique for thin LIBs based on ultrasonic guided waves. This method employed a piezoelectric transducer to excite guided wave signals and collects three-dimensional wave field data using a scanning laser Doppler vibrometer (SLDV). It represents the first application of ultrasonic guided waves for the non-destructive detection of the internal structure of LIBs and offers a novel approach for quality monitoring during battery production. Regarding the mechanical vibration characteristics of plastic-cased lithium batteries, Li et al. [18] developed a four-degree-of-freedom viscoelastic spring–mass model and measured the vibration velocity of the battery surface using LDV. The model demonstrated a frequency-domain amplitude fitting error of less than 11.36%, offering a theoretical framework for non-invasive assessment of battery health, particularly facilitating rapid diagnostics of retired batteries.

Although the two aforementioned methods eliminate the need for couplants, ACUT exhibits significant energy loss due to signal attenuation caused by acoustic impedance mismatch with air. This limitation restricts its penetration depth in thick battery cells. LDV, on the other hand, depends on an expensive optical system and is sensitive to the reflective properties of the battery surface. Therefore, these two methods are not fully applicable in complex industrial environments.

Most battery shells are made of aluminum or iron. Electromagnetic ultrasonic transducers (EMATs) can directly excite sound waves inside the battery through electromagnetic induction, enabling non-contact detection. This method can penetrate the battery shell without the need for couplants, significantly enhancing detection efficiency and environmental adaptability. Current research primarily focuses on defect detection in metal materials [19,20,21], thickness measurement [22,23], and related fields. However, its application to LIBs remains in the early stages. Li et al. [24] proposed a non-contact LIB state detection method based on EMAT. By developing a multi-layer porous battery finite element model, they analyzed the effects of ultrasonic guided wave dispersion characteristics and boundary reflections on the signal. Optimizing the EMAT magnet and coil structure increased the signal amplitude by a factor of 62.7. The results demonstrated that this technology can identify battery aging and uneven electrolyte infiltration, offering a new scheme for online safety monitoring of power batteries. The research presented in this paper aims to build a stable and reliable battery detection system by independently designing an EMAT and optimizing the matching of electromagnetic ultrasonic parameters with the characteristics of battery materials. This technology overcomes the physical limitations of traditional detection methods and provides real-time, non-destructive solutions for power battery health management. The main contributions of this paper are as follows:

- Based on the internal ultrasonic transmission characteristics of LIBs and the principle of electromagnetic ultrasonic excitation, an EMAT for battery detection is designed. This structure integrates magnets, coil configurations, and matching circuits. Finite element modeling and simulation methods are employed to optimize the static magnetic field strength and eddy current distribution density, significantly enhancing the transducer’s performance. By comparing the received signals of the probe before and after optimization, the impact of parameter optimization on the amplitude of the detection signal is quantitatively verified.
- Based on the optimized EMAT, a non-contact detection experimental system is built. A system reliability experiment is conducted to compare the detection results of the EMAT and PUT. By analyzing the performance differences in penetration ability and signal stability, it is confirmed that the proposed electromagnetic ultrasonic detection method is both reliable and applicable.
- Using the constructed electromagnetic ultrasonic system, a batch of retired LIBs with a nominal capacity of 270 Ah is tested through local point scanning, and their correlation with the remaining battery capacity is analyzed. The experimental results show that the ultrasonic signal amplitude (SA) of batteries with a capacity below 240 Ah is significantly lower than that of batteries with a capacity above 240 Ah, exhibiting an attenuation of 44%. These results confirm the feasibility of applying the system for rapid sampling inspection of large quantities of batteries and provide an effective method for the non-destructive and efficient evaluation of battery health status.

The structure of this paper is as follows: The Section 2 introduces the propagation characteristics of ultrasonic waves in LIBs and the excitation principles of electromagnetic ultrasonics. Additionally, it presents the design of the magnet structure, coil structure, and matching circuit for the electromagnetic ultrasonic probe. The Section 3 describes the finite element modeling process of the transducer, the optimization of specific parameters, and overview the construction of the experimental test system and the experimental setup. The Section 4 presents and discusses the simulation and experimental results. The Section 5 provides a summary of the entire paper.

## 2. Materials and Methods

### 2.1. Propagation Characteristics of Ultrasonic Wave in Batteries

An LIB is primarily composed of a cathode, an anode, a separator, an electrolyte, and an outer casing. The cathode and anode consist of porous electrodes, current collectors, conductive additives, and binders. The active material of the cathode is typically a lithium metal oxide, such as lithium cobalt oxide (LiCoO_2_) or lithium iron phosphate (LiFePO_4_), while the active material of the anode is usually a carbon-based material, such as graphite. During the charging process, lithium ions are extracted from the cathode material, migrate through the separator, and diffuse through the electrolyte into the anode material. Simultaneously, electrons flow from the cathode through the cathode current collector and external circuit to the anode current collector, completing the circuit. The discharge process is the reverse of the charging process.

Ultrasonic waves, defined as mechanical waves with frequencies exceeding 20 kHz, propagate through elastic media. The operational frequency of ultrasonic waves is a critical parameter that balances resolution and penetration depth. Higher frequencies generally offer better resolution for imaging fine details but suffer from increased attenuation, which limits their penetration into the medium. Conversely, lower frequencies experience less attenuation and can penetrate deeper into the material, albeit at the expense of reduced resolution. In battery inspection, typical ultrasonic frequencies range from a few hundred kilohertz to several megahertz, providing sufficient penetration to examine internal structures across the thickness of common pouch or prismatic cells while maintaining the sensitivity needed to detect features such as electrode layers, porosity, and gas accumulation.

In solids, they travel in two modes: longitudinal waves, where particle vibration is parallel to the direction of propagation, and shear waves, where particle vibration is perpendicular to the direction of propagation. Longitudinal waves can propagate through solids, liquids, and gases, whereas shear waves exist exclusively in solids.

**Figure 1:**
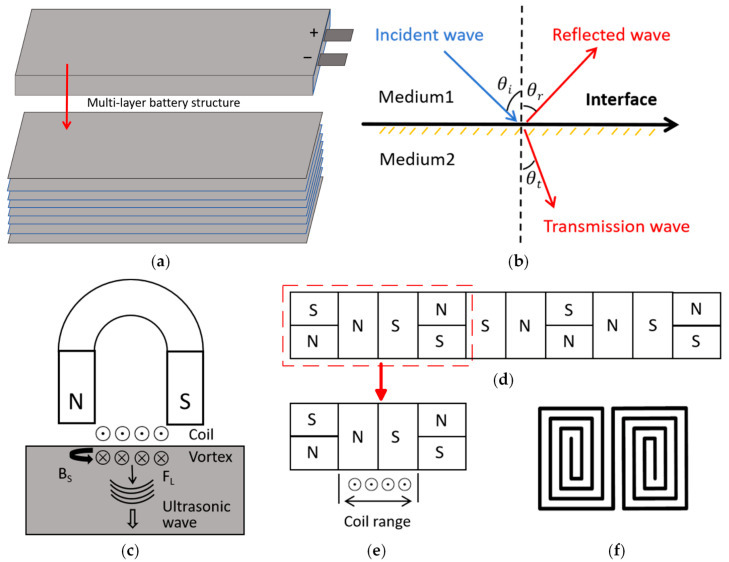
(**a**) Multi-layer structure diagram of battery; (**b**) the reflection and refraction of ultrasonic waves passing through the interface between two media; (**c**) the principle of electromagnetic ultrasonic longitudinal wave generation; (**d**) schematic diagram of a Halbach array magnet; (**e**) one-cycle Halbach magnet and coil; (**f**) butterfly coil.

LIB electrodes consist of densely packed active material particles. When ultrasonic waves propagate through the contact points between particles, signal attenuation occurs due to reflection and refraction at irregular interfaces, as well as frictional dissipation between particles. In multilayer battery structures (see Figure 1a), ultrasonic wave propagation is governed by the wave equation [25]:

(1) $$\rho \frac{\partial^{2}u}{{\partial t}^{2}}=\mu \nabla^{2}u+\left(\lambda +\mu \right)\nabla \left(\nabla \cdot u\right)-d\frac{\partial u}{\partial t}$$

where $u(x_{1},x_{2},x_{3})$ is the displacement field at the coordinate $x(x_{1},x_{2},x_{3})$, μ and λ represent the Lamé constants, ρ is the density of the test sample, and d is the damping coefficient, which is related to the damping ratio. In this equation, we omit F to represent the propagation of waves in the medium after the excitation source has ended.

When a battery undergoes multiple charge–discharge cycles or experiences internal electrolyte decomposition, substantial gas generation may occur. This leads to a significant impedance mismatch at gas–solid interfaces, which markedly increases ultrasonic attenuation. As ultrasonic waves propagate across the interface between two media, reflection and transmission phenomena occur at the boundary, as illustrated in Figure 1b. In battery ultrasonic testing, signals typically impinge perpendicularly on each material layer (i.e., normal incidence). Under this condition, where $\theta_{i}=\theta_{r}=\theta_{t}=0$, the reflection coefficient $R_{I}$ and transmission coefficient $T_{I}$ are calculated as follows:

(2) RI=−Z2Z1−1Z2Z1+12=Z2−Z12Z2+Z12

(3) TI=4Z2Z1Z2Z1+12=4Z1·Z2Z1+Z22

where $Z_{1}$ and $Z_{2}$ are the acoustic impedances of the media on either side of the interface, and Z is calculated as the product of the medium’s density and the sound velocity.

The acoustic parameters of several common substances in batteries are presented in Table 1. As shown in Table 1, the acoustic impedance of gases is significantly lower than that of solid materials. This disparity causes strong reflection of ultrasonic signals at gas–liquid and gas–solid interfaces, leading to a sharp attenuation of ultrasonic signal energy in regions where gas is present. Therefore, by analyzing the attenuation of the time-domain peak value of the transmitted signal, non-destructive detection of gas formation inside the battery can be achieved.

### 2.2. Electromagnetic Ultrasonic Principle

The electromagnetic ultrasonic transducer consists of a high-frequency coil, a permanent magnet, and a test specimen. The excitation mechanisms include the Lorentz force and the magnetostrictive effect. For non-ferromagnetic aluminum-plastic film-encapsulated LIBs, only the Lorentz force-dominant mechanism needs to be considered. When the coil is connected to a high-frequency alternating current, it generates not only a dynamic magnetic field $B_{d}$ within the coil assembly but also induces eddy currents $J_{e}$ on the surface of the specimen that oppose the applied current. The expression for the eddy current is as follows:

(4) $$J_{e}=-\sigma \frac{\partial A}{\partial t}$$

where A represents the vector magnetic potential and σ represents the electrical conductivity.

The induced eddy currents that are not parallel to the direction of the magnetic field generate Lorentz force $F_{S}$ and $F_{d}$ under the influence of the applied magnetic field $B_{S}$ and the dynamic magnetic field $B_{d}$. As a result, the surface of the test piece vibrates at a high frequency, thereby stimulating ultrasonic waves. This phenomenon is known as the generation of electromagnetic ultrasonic waves. The expressions for the respective forces and the total Lorentz force are as follows:

(5) $$F_{S}=J_{e}\times B_{S}$$

(6) $$F_{d}=J_{e}\times B_{d}$$

(7) $$F_{L}=F_{S}+F_{d}$$

The electromagnetic ultrasonic excitation mode is determined by the direction of the bias magnetic field on the specimen’s surface. A horizontal magnetic field excites longitudinal waves, while a vertical magnetic field excites transverse waves. Since electromagnetic ultrasound is only suitable for conductive media, this study employs a horizontal magnetic field to excite longitudinal waves for detecting aluminum-shell packaged lithium batteries (whose conductivity meets the requirements). The principle is illustrated in Figure 1c.

### 2.3. EMAT Design

#### 2.3.1. Halbach Array Magnet Structure

To excite longitudinal waves on the surface of the battery, the magnetic field in the x-y plane must be oriented perpendicular to the direction of the high-frequency coil current. In this paper, a novel arrangement of permanent magnets, such as the Halbach array, is employed, as shown in Figure 1d. This configuration arranges permanent magnets with varying magnetization directions in a specific sequence. According to the superposition principle, the magnetic flux lines on one side of the array reinforce each other, while those on the opposite side largely cancel out. Consequently, the magnetic field on one side of the array is significantly enhanced, and on the other side, it is substantially weakened. This design achieves the generation of the strongest possible magnetic field using the fewest magnets.

This experiment uses a linear Halbach permanent magnet. The magnetic induction intensity on the side of the strong magnetic field of the linear Halbach magnet is:

(8) $$B_{0}=B_{r}\left[1-e^{-kd}\right]\sin\left(\frac{\pi }{m}\right)∕\left(\frac{\pi }{m}\right)$$

(9) $$B_{y}=B_{0}\sin\left(ky\right)e^{-kz}$$

(10) $$B_{z}=B_{0}\cos\left(ky\right)e^{-kz}$$

where k is the wave number of the linear Halbach permanent magnet, k=2π/λ, λ is the magnet wavelength, m is the number of magnetic blocks of each wavelength. $B_{0}$ is the magnetic induction intensity on the surface of the magnet on the side of the strong magnetic field. $B_{y}$ and $B_{z}$ are the components of the magnetic induction intensity along the y and z directions, respectively, on the side with the strong magnetic field of the linear Halbach magnet.

The static magnetic induction intensity on the surface of a Halbach permanent magnet is:

(11) $$B_{sz0}=B_{0}\cos\left(ky\right)$$

The static magnetic flux density at a distance L from the surface of the magnet is:

(12) $$B_{szL}=B_{0}\cos\left(ky\right)e^{-kL}$$

(13) $$B_{syL}=B_{0}\sin\left(ky\right)e^{-kL}$$

In the basic structure of the electromagnetic ultrasonic transducer, the coil is primarily positioned in the central area. Therefore, it is only necessary to ensure that the magnetic field enhancement covers the coil’s range. This paper adopts a single-cycle arrangement, as shown in Figure 1e. This structure not only effectively increases the magnetic field strength in the eddy current region beneath the coil but also conserves magnet material and reduces the space occupied by the magnet.

#### 2.3.2. Butterfly Coil

For the excitation of electromagnetic ultrasonic waves, commonly used coil structures include tortuous coils, spiral coils, and butterfly coils. Different shapes of permanent magnets and coils with various winding methods can excite different types of ultrasonic waves. In this paper, based on the unilateral magnetic field enhancement characteristics of the Halbach permanent magnet, the magnetic force direction of the magnet is oriented horizontally. To excite longitudinal waves, the coil structure selected is the butterfly coil shown in Figure 1f. Because the butterfly coil is arranged in the central area beneath the magnet, it ensures consistent magnetic field direction through the symmetrical cyclotron structure, effectively reducing magnetic circuit interference. Additionally, the coil’s equidistant expansion characteristics allow flexible control of the effective number of turns per unit width by adjusting the spacing between turns. This design balances electromagnetic performance and space utilization, creating a collaborative structure that is highly compatible with the Halbach magnetic field distribution.

### 2.4. Match Circuits

The low energy conversion efficiency of EMATs causes significant attenuation of ultrasonic echo signals and a deterioration of the signal-to-noise ratio (SNR). This limitation severely restricts their use in engineering applications for non-destructive testing. Within safety thresholds, increasing the excitation current amplitude can significantly enhance the amplitude of the ultrasonic echo signal. The transducer coil designed in this paper exhibits notably low resistance characteristics (typical value: several ohms), which causes a significant impedance mismatch with the internal resistance (50 Ω) of the conventional excitation source. This mismatch results in a substantial reduction in power transmission efficiency. To achieve a significant increase in the amplitude of the excitation current and ensure maximum power transmission, an effective matching mechanism between the load impedance and the internal resistance of the excitation source must be established.

**Figure 2:**
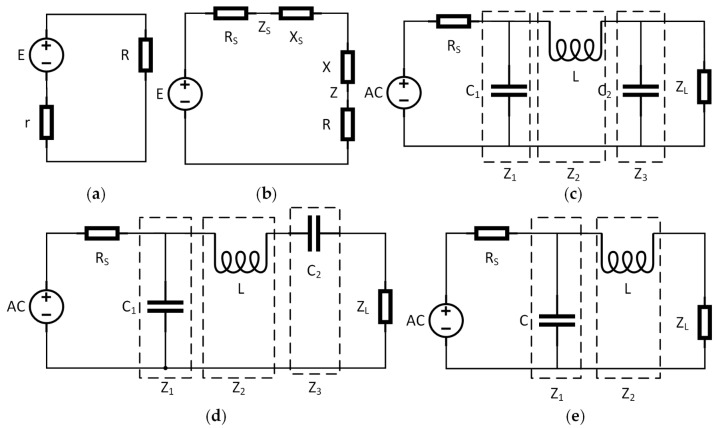
(**a**) Pure resistive circuit; (**b**) impedance-containing circuit; (**c**) π -type impedance matching network; (**d**) τ-type impedance matching network; (**e**) L-type impedance matching network.

As shown in Figure 2a, in a purely resistive circuit, to maximize the output power, the load resistance must be equal to the internal resistance of the excitation source. The output power is given by:

(14) $$P=I^{2}R={\left(\frac{E}{R+r}\right)}^{2}=\frac{E^{2}}{4r+{\left(R-r\right)}^{2}/R}$$

In the circuit with impedance, as shown in Figure 2b, the resistance is represented as a vector, combining resistance and reactance according to the formula $Z=\sqrt{R^{2}+{\left(X_{L}-X_{C}\right)}^{2}}$. For the detection process of the EMAT, this paper matches the signal input end—that is, the excitation source—with the excitation coil. The impedance of the excitation source can be expressed as:

(15) $$Z_{i}=R_{i}+jX_{i}$$

The impedance of the excitation coil can be expressed as:

(16) $$Z_{E}=R_{E}+jX_{E}$$

where $R_{i}$, $X_{i}$, $R_{E}$, and $X_{E}$ are real numbers. The output power reaches its maximum when the following equations are satisfied:

(17) $$R_{i}=R_{E}$$

(18) $$X_{i}=-X_{E}$$

According to the combination method, there are three common impedance matching networks: the π-type matching network, the τ-type matching network, and the L-type matching network. The appropriate use of each circuit depends on the actual working conditions. The three matching methods are described below.

1. π-type matching network

As shown in Figure 2c, this network corrects impedance mismatches from high to low within a circuit. It is widely employed in broadband matching applications, particularly in broadcasting, wireless communication, and related fields. It consists of either two inductors and one capacitor or two capacitors and one inductor, requiring three impedance components. Here, R_S_ represents the internal resistance of the excitation source; Z_1_, Z_2_, and Z_3_ denote the equivalent impedances from the input to the output end, respectively; and Z_L_ is the load impedance. For maximum energy transfer efficiency, Z_3_ and Z_L_ must satisfy the conjugate matching condition.

2. τ-type matching network

As shown in Figure 2d, the network also requires a three-part impedance and is suitable for broadband impedance matching. It offers design flexibility, particularly in matching the load impedance to the source impedance, allowing it to accommodate large impedance variations while maintaining high power transmission efficiency.

3. L-type matching network

As shown in Figure 2e, the L-shaped impedance matching network consists of only one series element and one parallel element, making it structurally simpler. To achieve maximum power transfer efficiency, Z_2_ and Z_L_ must satisfy the conjugate matching conditions. However, compared to the other two methods, this approach has fewer degrees of freedom: the imaginary part of the load cannot be adjusted, and the Q factor is solely determined by the impedance transformation ratio and the excitation frequency.

After adding the impedance matching circuit, the overall impedance of the coil is given by the following formula:

(19) $$Z=jX_{a}/\left[R_{E}+j\left(X_{b}+X_{E}\right)\right]$$

To simplify the formula, it is necessary to satisfy the conjugate matching condition with the excitation source to achieve maximum output power. This can be expressed as follows:

(20) $$X_{a}=-\frac{{R_{i}}^{2}+{X_{I}}^{2}}{X_{i}+QR_{i}}$$

The definition of Q is as follows:

(21) $$Q=\pm \sqrt{\frac{R_{i}}{R_{E}}\left[1+{\left(\frac{X_{i}}{R_{i}}\right)}^{2}\right]-1}$$

After the comprehensive analysis above, considering that the impedance of the transducer coil designed in this paper differs significantly from that of the excitation source, both L-type and τ-type matching circuits are viable options. However, the matching network must accommodate the limited cavity space of the 3D-printed probe. The L-type circuit requires only two passive components, reducing the board area by 33% compared to the three-component configuration of the τ/π-type network. Its compact structure is more conducive to maintaining high-frequency signal integrity. In addition, the tuning degree of freedom analysis shows that although the τ/π-type network offers additional degrees of freedom, it can result in multiple convergent solutions. In contrast, the single-solution characteristic of the L-type network ensures the uniqueness of the matching parameters and reduces the complexity of engineering debugging. Therefore, the L-type matching circuit is ultimately selected in this paper.

## 3. EMAT Simulation and Experiment

### 3.1. Subsection

In this paper, the finite element software COMSOL Multiphysics 6.2 is used to establish a two-dimensional model of an EMAT, and a multiphysics finite element simulation analysis of its operating process is conducted.

According to the analysis in the previous chapter, the probe design model primarily consists of three components: a permanent magnet, a coil, and a test piece. The test piece is originally intended to be a battery. Since the battery’s surface is coated with aluminum, it can be replaced by aluminum or aluminum alloy material in the simulation. The magnetic field strength generated by the bias static magnetic field is calculated using the magnetic field without current in the AC/DC module. The magnetic field module is then employed to determine the dynamic magnetic field strength and the eddy current density induced in the coil by the alternating current. The solid mechanics module, within the structural mechanics module, incorporates the Lorentz force as a body load. Under the influence of the Lorentz force, the particle begins to vibrate, and the resulting displacement from this vibration propagates as an ultrasonic wave.

**Figure 3:**
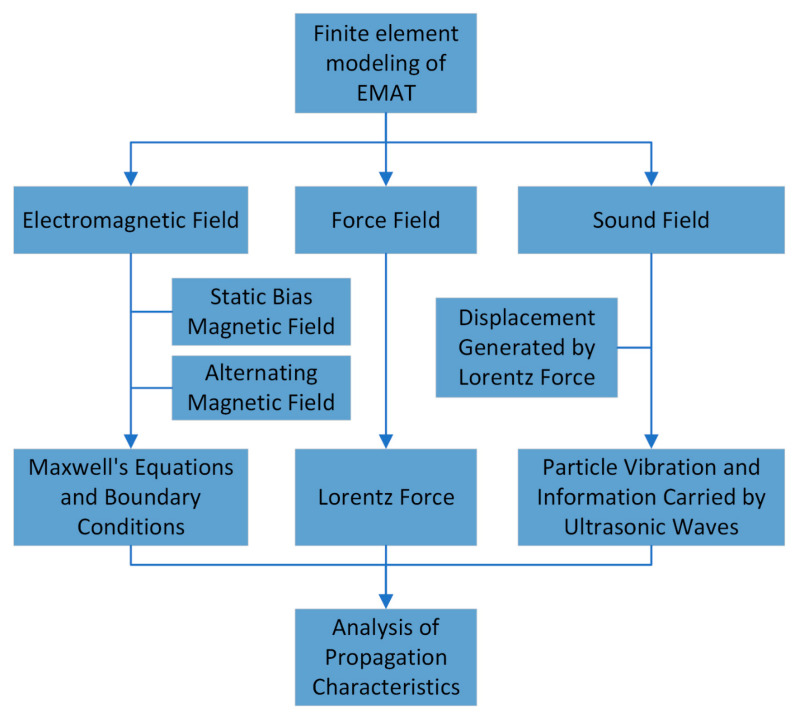
Finite element simulation analysis process.

The simulation flowchart is shown in Figure 3. First, the dimensions of the two-dimensional model are selected. Next, the required physical layer is chosen, the geometric model is constructed, and the coupling of each physical field is configured.

**Figure 4:**
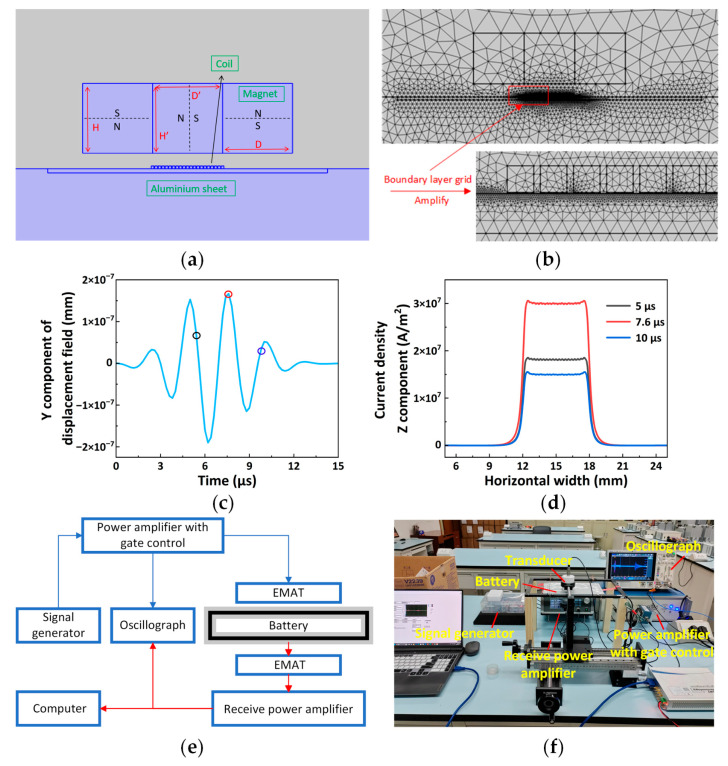
(**a**) Two-dimensional finite element model of an EMAT; (**b**) diagram of the finite element model grid subdivision results; (**c**,**d**) the excitation five-peak wave signal for a specific coil parameter and the corresponding eddy current density at several time points; (**e**) schematic diagram of the experimental device connections; (**f**) physical picture of the ultrasonic testing system.

The simulation model is shown in Figure 4a. The permanent magnet used is an N52 neodymium iron boron magnet, sourced from Yijia Magnet, Ganzhou, China. It has a conductivity of 7.1426 × 10^5^ S/m, a relative permeability of 1.05, and a residual magnetic flux density of 1.44 T. The tested sample has a Young’s modulus of 7.1 × 10^10^ Pa, a Poisson’s ratio of 0.25, a conductivity of 3.775 × 10^7^ S/m, and a density of 2810 kg/m^3^. The transducer coil is constructed using a butterfly winding. The number of central turns, wire width, height, line spacing, and the lift-off distance between the bottom of the permanent magnet and the upper surface of the test piece are specified. Since the current direction in the central part of the butterfly coil is uniform, this middle section serves as the primary working area, allowing the coil sections on both sides to be omitted in the simulation. The excitation coil is driven by an alternating current consisting of a sine wave modulated by a Hanning window, with a 5-cycle center frequency of 400 kHz. The Hanning window, a smooth bell-shaped curve that tapers to zero at both ends, is applied to reduce spectral leakage by ensuring the signal starts and ends smoothly, rather than abruptly.

After establishing the geometric model and physical field, the mesh is generated. The mesh can be composed of free triangular elements, free quadrilateral elements, or mapped elements. Users can specify the maximum and minimum element sizes for the mesh. In this model, each structure utilizes a free triangular mesh. According to the role of each component in the coupling field, a hierarchical discretization strategy is implemented. A sparse grid structure is employed in the static magnetic field conduction region (such as the air medium) to reduce computational load, while the high-frequency physical field region (such as the aluminum specimen exhibiting eddy current skin effect and the ultrasonic propagation path) is locally refined. To improve the calculation accuracy of the field quantities, boundary layer grids are constructed at key locations, such as the aluminum-permanent magnet interface and the coil edge, effectively mitigating gradient distortion of the field quantities. The result of the division is shown in Figure 4b. Different solvers are assigned according to the physical fields of the respective modules. The magnetic field current-free module uses a steady-state solver without specifying a time step. Both the magnetic field and solid mechanics modules are solved using a transient solver. The time step for the transient solver is 0.2 µs, and the total simulation time is 15 µs.

### 3.2. Parameter Optimization

#### 3.2.1. Magnet Parameter Optimization

As shown in Figure 4a, the Halbach configuration magnet consists of two parts: two magnets with identical left–right symmetrical specifications but opposite magnetic pole directions, and a magnet in the middle with its magnetic field oriented horizontally to the left. Both magnets are magnetized through their thickness. This configuration generates a horizontal magnetic field at the eddy current location. According to Equation (5), increasing the intensity of the horizontal static magnetic field effectively enhances the Lorentz force, thereby improving the strength of the excitation signal. However, while increasing this field, the influence of the vertical static magnetic field intensity should be minimized, as the eddy current can induce shear waves under the effect of the vertical static magnetic field, which compromises the purity of the signal.

The original parameters of the ultrasonic transducer are set as follows: the number of turns of the coil center is N = 21; the wire width is w = 0.2 mm; the height is l = 0.2 mm; the line spacing is d = 0.05 mm; the distance between the bottom of the permanent magnet and the upper surface of the test piece is h = 1.2 mm; the distance between the coil and the upper surface of the test piece is s = 0.1 mm; the width of the left and right magnets is D = 5 mm; the height is H = 5 mm; the width of the central magnet is D′ = 5 mm; and the height is H′ = 5 mm.

Therefore, the optimization of the static magnetic field strength can be conducted using the following approach (control variable method):

1. Change the width of the middle magnet while keeping the widths of the left and right magnets unchanged;
2. Change the width of the left and right magnets while keeping the width of the middle magnet unchanged;
3. Change the height of the permanent magnet;
4. Increase the number of magnets superpositions to further enhance the strength of the static magnetic field.

#### 3.2.2. Coil Parameter Optimization

From Formula (5), it is evident that increasing the eddy current density effectively enhances the Lorentz force, thereby improving the intensity of the excitation signal. The coil parameters can be adjusted in the following aspects: the number of central turns, the wire width, the coil height, and the line spacing. As shown in Figure 1c, the coil current direction is perpendicular to the plane of the paper, and the induced eddy current flows in the opposite but parallel direction. However, when the coil parameters remain unchanged, the eddy current density varies with the signal (Figure 4c,d). The eddy current densities at three different times, shown in Figure 4d, are highlighted by the three different colored circles in Figure 4c. In the subsequent parameter adjustments, the curves corresponding to the highest eddy current density are compared.

Therefore, the coil parameters can be optimized using the following aspects (control variable method):

1. The number of central turns of the coil;
2. Coil diameter;
3. The line spacing of the coil.

#### 3.2.3. Spatial Configuration Parameters Optimization

1. The distance between the bottom of the magnet and the surface of the measured object remains constant, while the distance between the bottom of the coil and the surface of the measured object varies;
2. The distance between the bottom of the coil and the surface of the measured object remains unchanged, while the distance between the bottom of the magnet and the surface of the measured object varies.

### 3.3. Experimental Design

#### 3.3.1. Ultrasonic Testing System Design

The connection diagram of the electromagnetic ultrasonic testing device is shown in Figure 4e. The signal generator emits a five-peak wave signal with a center frequency of 400 kHz, along with a square wave timing signal modulated by a Hanning window. The square wave timing signal controls the power amplifier’s on/off state. After the sinusoidal signal is amplified by the power amplifier, it passes through the impedance matching circuit, and then the electromagnetic ultrasonic signal is excited on the battery specimen by the optimized emission transducer. The electromagnetic ultrasonic signal propagates through the battery. After traversing the complex propagation path, the ultrasonic signal is received by the probe at the opposite end of the battery. The signal is first adjusted by an impedance matching circuit, and then its amplitude is amplified by a receive power amplifier. Finally, the data is displayed and collected in real time using an oscillograph. After band-pass filtering, the characteristic parameter, signal amplitude (SA), is extracted to identify battery defects. The physical object of the ultrasonic testing system is shown in Figure 4f.

#### 3.3.2. Transducer Reliability Experiment Design

Using the designed ultrasonic detection system, the optimized electromagnetic ultrasonic transducer is used to detect standard aluminum blocks measuring 4 cm and 6 cm, respectively, and the received signals are recorded.

Using the same excitation signal, the two probes are replaced with a 500 kHz piezoelectric ultrasonic probe. The stability and noise of the received signals from both probes are observed, and their flight times are compared. Because the piezoelectric ultrasonic probe has a higher energy conversion efficiency, the gain of the gated amplifier and the post-amplifier needed to be reduced.

**Figure 5:**
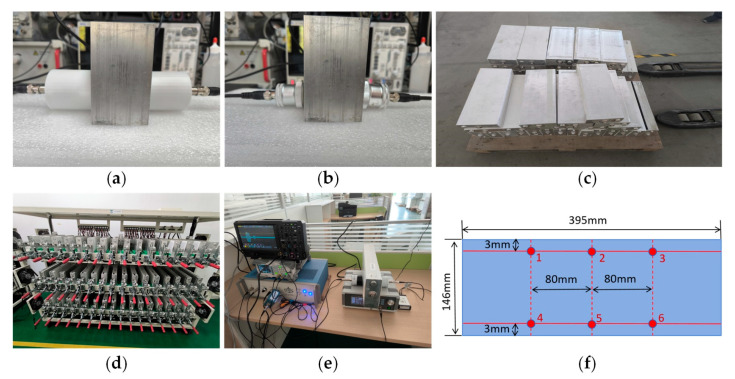
(**a**) Electromagnetic ultrasonic testing of a 6 mm aluminum block; (**b**) piezoelectric ultrasonic testing of a 6 mm aluminum block; (**c**) the 48 selected retired batteries; (**d**) the battery is divided into charge and discharge piles for capacity operation; (**e**) point-scanning experimental conditions; (**f**) measurement point selection.

The working condition diagrams of the electromagnetic ultrasonic transducer and the PUT for detecting a 6 mm standard aluminum block are shown in Figure 5a and Figure 5b, respectively.

#### 3.3.3. Application in Retired Batteries

For a large number of batteries in the factory, performing a comprehensive two-dimensional surface scan would be very time-consuming. Therefore, several specific points on each battery can be selected, and the measured time-domain peak values at these points are averaged. This parameter is then used to approximate the battery’s condition and assist the company in quickly categorizing retired battery types.

In this study, a total of forty-eight (48) retired lithium iron phosphate (LFP) batteries from BYD Company Limited, Shenzhen, China, were tested. The specific model is FADMO7315B, produced by Huizhou BYD Battery Co., Ltd., Huizhou, China. These batteries were originally used in electric vehicles (EVs). Prior to testing, the batteries had an actual service life ranging from approximately 4 to 9 years. Battery lifespan is influenced by factors such as usage patterns, charging habits, and environmental conditions.

As shown in Figure 5c, each with a standard capacity of 270 Ah and a thickness of 58 mm, are selected. Since their capacity varies after many years of use, all 48 batteries are first subjected to a series of tests: constant current discharge, constant current and constant voltage charging, constant current discharge, constant current and constant voltage charging, and constant current discharge (as shown in Figure 5d). Each step is followed by a 30 min rest period. The aforementioned operating conditions are all configured by the host computer software. The 30 min standby period allows the batteries to stabilize and cool down, preventing potential thermal runaway before proceeding to the next process. The final tested capacity is considered the actual capacity of the battery.

When the batch of batteries has been completed, each battery is labeled with its actual capacity. Due to the accumulation of dust on the surfaces of batteries that have been in use for many years, they are cleaned before testing to ensure consistent coupling of the EMAT. Then, the battery measurement system performs point scanning operations on each battery individually. The experimental test setup is shown in Figure 5e, and the selected measurement points are shown in points 1 to 6 of Figure 5f.

## 4. Results and Discussion

### 4.1. Simulation Results and Analysis

#### 4.1.1. Result of Magnet Parameter Optimization

**Figure 6:**
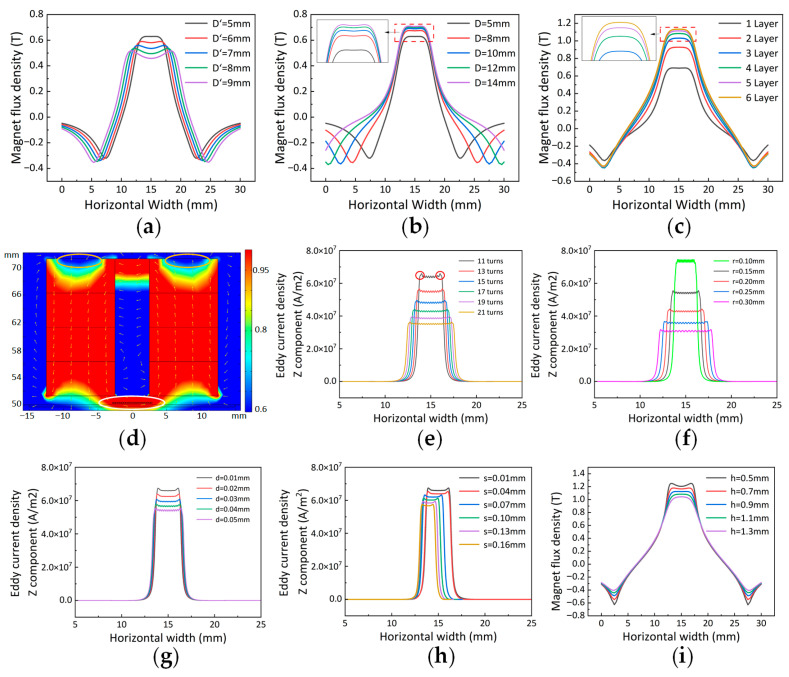
(**a**) The x component of the magnetic flux density on the battery surface at different values of H’; (**b**) the x component of the magnetic flux density on the battery surface at different values of D; (**c**) the x component of the magnetic flux density on the battery surface at different values of height; (**d**) magnetic flux density and intensity image; (**e**) eddy current density at different numbers of coil turns; (**f**) eddy current density for various wire diameters; (**g**) eddy current density at different line spacings; (**h**) eddy current density at different distances between the coil and the upper surface of the measured object; (**i**) the x component of the magnetic flux density at different distances between the magnet and the upper surface of the measured object.

For adjustment 1, as shown in Figure 6a, it can be observed that with the increase in the width of the intermediate magnet, the horizontal component of the magnetic flux density continuously decreases, and the central peak becomes increasingly uneven (exhibiting a downward depression). This results in a failure to provide a uniform horizontal magnetic field at the coil position. Therefore, the central magnet with a width of 5 mm is selected.

For adjustment 2, as shown in Figure 6b, with the increase in the width of the left and right magnets, the horizontal magnetic field strength of the permanent magnet at the battery surface gradually increases. However, when the width exceeds 10 mm, the growth rate of the magnetic field strength slows down. Considering the size of the permanent magnet and the portability of the subsequent probe, a width of D = 10 mm for the left and right magnets is selected.

Considering that increasing the number of magnets also changes the height of the permanent magnet, adjustments 3 and 4 are made simultaneously. The height of each layer is 5 mm. Control the thickness of the middle magnet and the width of the left and right magnets accordingly. As shown in the central region of the curve of Figure 6c, the horizontal magnetic field strength of the permanent magnet at the battery surface gradually increases with the number of superimposed layers. However, when the number of layers exceeds four, the growth rate of the magnetic field strength does not significantly slow down. Considering the size of the permanent magnet and the portability of the subsequent probe, four layers of magnet superposition are selected.

Therefore, the permanent magnet uses the following parameters: the width of the left and right magnets is D = 10 mm, the height is H = 5 mm, and four layers are stacked; the width of the central magnet is D′ = 5 mm, and the height is H′ = 20 mm.

The distribution of the magnetic flux density magnitude is shown in Figure 6d. Specifically, a high flux density occurs near the coil (indicated by the white ring), while a low flux density appears on the upper surface of the permanent magnet (indicated by the yellow ring). This observation not only aligns with the optimization of the EMAT but also confirms the feasibility of the Halbach configuration from this perspective.

#### 4.1.2. Result of Coil Parameter Optimization

For adjustment 1, as shown in Figure 6e, when the number of turns decreases, the eddy current density becomes stronger and more concentrated. However, within this concentrated area, the density increases toward both sides, meaning the eddy current density on the battery surface beneath the coil is uneven. Specifically, the density indicated by the red circle, is higher, which adversely affects the purity of the longitudinal wave signal (since variations in eddy current density result in differences in the Lorentz force excitation intensity of the longitudinal wave). Meanwhile, although the eddy current density decreases as the number of turns increases, the ultrasonic vibration intensity resulting from the combined action of multiple coils is stronger than that with fewer turns, producing a purer longitudinal wave. Based on the above, the coils can cover the central high magnetic field strength area. Accordingly, the multi-turn coil should be positioned to cover the central region of high magnetic flux density.

For adjustment 2, as shown in Figure 6f, when the wire diameter decreases, the eddy current density becomes stronger and more concentrated. However, using excessively fine copper wire increases the difficulty of winding and the likelihood of damage. Considering these factors comprehensively, a wire diameter of r = 0.11 mm is selected.

For adjustment 3, as shown in Figure 6g, it can be observed that as the line spacing decreases, the eddy current density becomes stronger and more concentrated. In practice, the outer layer of copper wire is typically coated with insulating paint, allowing the line spacing to be minimized as much as possible during winding.

#### 4.1.3. Result of Spatial Configuration Parameters Optimization

As shown in Figure 6h, when the magnet-to-surface spacing is constant, the surface magnetic field strength remains stable. However, increasing the coil-to-surface spacing causes a decrease in eddy current density, thereby reducing the SNR of the Lorentz force measurement. Therefore, in the actual experiment, the coil is positioned close to the battery surface to enhance the SNR.

Figure 6i shows that as the distance between the magnet and the battery surface increases, the horizontal magnetic field strength of the permanent magnet at the battery surface gradually decreases. Therefore, in the actual experiment, the magnet should be positioned as close as possible to the battery surface.

#### 4.1.4. The Final Parameter Structure of the EMAT

Based on the analysis above, the final optimized parameters for the permanent magnet and coil structure are summarized in Table 2.

**Figure 7:**
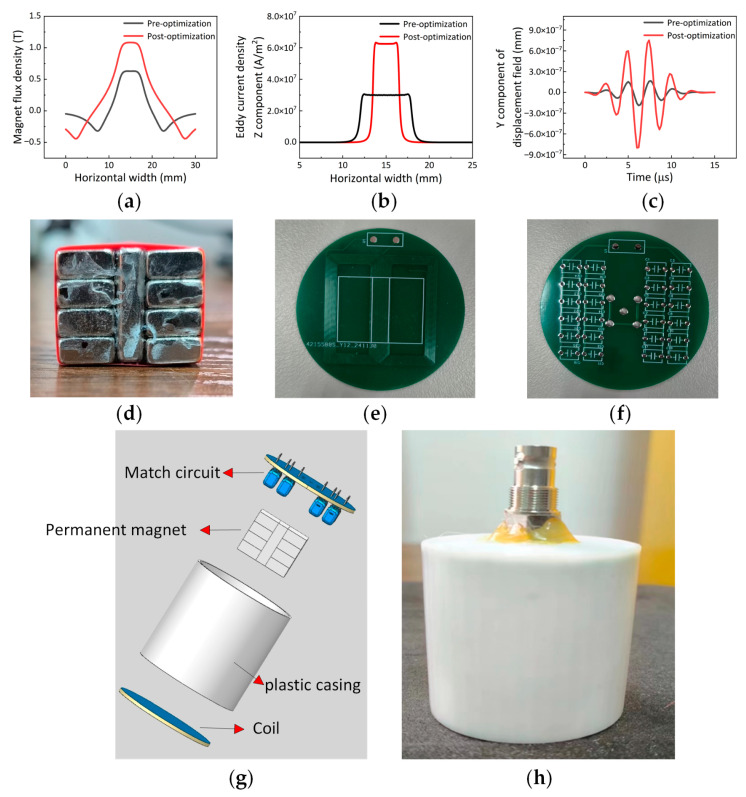
(**a**) Horizontal magnetic field intensity before and after optimization; (**b**) eddy current density in the skin layer before and after optimization; (**c**) excitation signals before and after optimization; (**d**) permanent magnet; (**e**) butterfly coil; (**f**) match circuit; (**g**) diagram of the overall structure of the transducer; (**h**) transducer physical map.

The final optimized Halbach configuration electromagnetic ultrasonic longitudinal wave transducer exhibits a horizontal magnetic field intensity on the specimen surface that is approximately twice as strong. Additionally, the eddy current density in the skin layer increases by about 3.1 times, and the amplitude of the excitation signal rises by approximately four times. The comparison diagrams are shown in Figure 7a–c. The physical diagrams of the permanent magnet, coil, and matching circuit of the transducer are shown in Figure 7d–f. The overall structure of the transducer is shown in Figure 7g, and the physical image of the transducer is shown in Figure 7h.

### 4.2. Transducer Reliability Analysis

**Figure 8:**
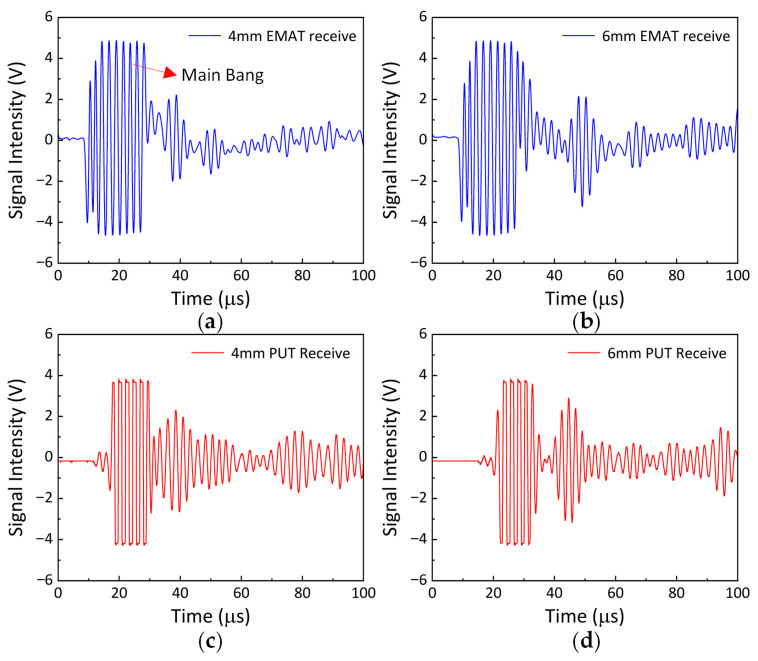
(**a**–**d**) The signals from 4 mm and 6 mm standard aluminum blocks are detected using electromagnetic ultrasonic and piezoelectric ultrasonic methods.

As shown in Figure 8a–d, both electromagnetic ultrasonic testing and piezoelectric ultrasonic testing exhibit significant and identifiable characteristic response curves in terms of signal feature analysis. The two methods demonstrate a high degree of consistency in the distribution of characteristic regions. However, due to the higher excitation voltage of electromagnetic ultrasound and the greater amplification of the received signal, the background noise level in the acquired signal is relatively elevated. Further calculations reveal that the Time of Flight (ToF) data in Figure 8a–d are 12.4 µs, 18.69 µs, 13.69 µs, and 18.17 µs, respectively. The time-of-flight results measured by electromagnetic and piezoelectric ultrasound show good correspondence.

Based on the correlation between the signal characteristics and flight time, this study confirms the feasibility of using electromagnetic ultrasonic technology to detect battery status.

### 4.3. Application Analysis in Retired Batteries

**Figure 9:**
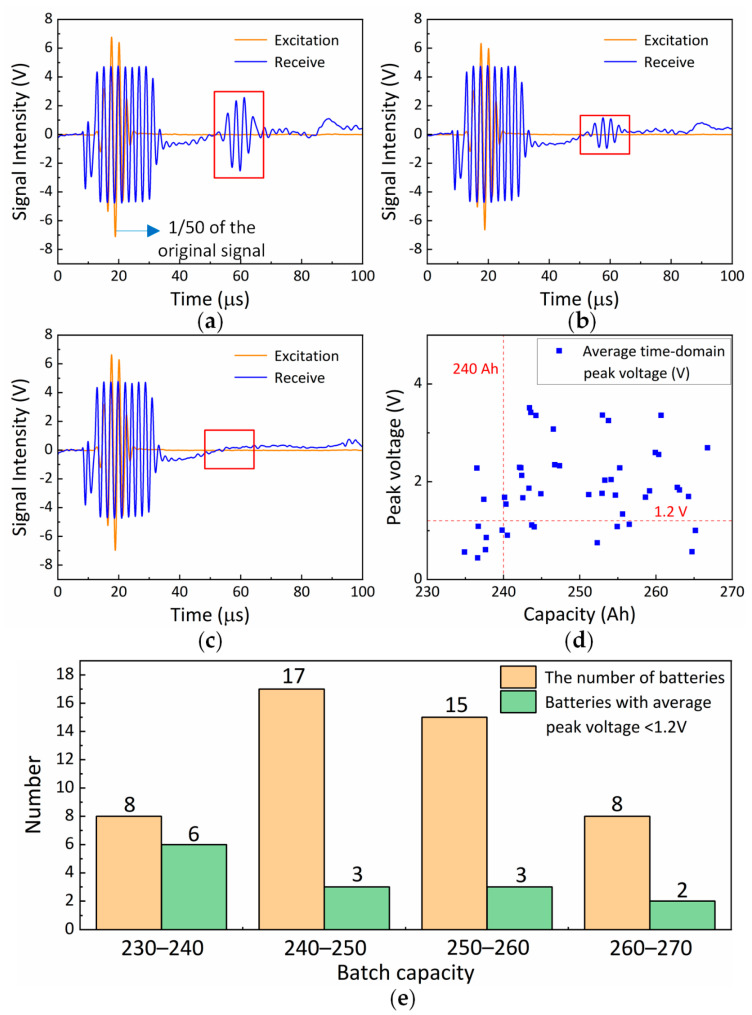
(**a**–**c**) Ultrasonic excitation signals received at three different points; (**d**) battery capacity and average time-domain peak scatter plot; (**e**) number of batteries and subset with peak voltage below 1.2 V across capacity ranges.

As shown in Figure 9a–c, the waveforms after band-pass filtering of ultrasonic data at three different points are presented. The excitation signal (yellow waveform) is attenuated by a factor of 50, while the received signal is shown as the blue waveform. Among them, Figure 9a represents a measurement point with a capacity of 266 Ah, while Figure 9c corresponds to a measurement point with a capacity of 234 Ah. To extract the time-domain peak, the effective echo signal between 50 µs and 75 µs is selected. This time window is determined based on the known thickness of the battery casing and the typical sound velocity within the lithium-ion cell, ensuring it captures the first significant through-thickness transmission wave following the initial pulse and before any confounding multiple reflections. This temporal gate is consistently applied across all batteries to ensure comparability of the extracted features. Figure 9a,b display clear received signals (highlighted by the red boxes), whereas Figure 9c shows almost no received signal.

Based on 288 sets of ultrasonic testing data (6 measurement points per battery) collected from 48 retired battery samples, the time-domain peak feature is extracted from each dataset as the key indicator. Firstly, the received signal is enveloped by applying the Hilbert transform. Then, the maximum value of the envelope is determined within the selected time window; this value represents the time-domain peak. To characterize the overall state of each single cell, the arithmetic mean of the time-domain peaks at the six measurement points is calculated to approximate the ultrasonic response characteristics of the cell. The resulting scatter plots of battery capacity versus peak voltage are shown in Figure 9d.

The samples are divided into four groups (230–240 Ah, 240–250 Ah, 250–260 Ah, and 260–270 Ah) based on capacity intervals. As shown in Figure 9d, most average time-domain peaks of batteries with capacities between 230 and 240 Ah are below 1.2 V, whereas most batteries with capacities exceeding 240 Ah have average time-domain peaks above 1.2 V. Figure 9e presents a more intuitive bar chart. Table 3 presents the number of batteries within each capacity range, the average time-domain peak, and its standard deviation for all batteries in each capacity group.

Statistical analysis reveals that the mean time-domain peak value of the 230–240 Ah group is significantly lower than those of the other three groups. This reduction is substantial, exceeding 40% compared to the means of the higher-capacity groups. In contrast, no statistically significant differences are found among the mean values of the 240–250 Ah, 250–260 Ah, and 260–270 Ah groups, and no gradient change with increasing capacity is observed. These observations suggest that batteries with a capacity below 240 Ah may experience electrolyte drying or an increase in air gaps due to aging, which significantly reduces ultrasonic propagation efficiency, thereby lowering the time-domain peak.

Based on our findings and a review of the existing literature, we propose that gas evolution during battery aging is the most probable primary factor contributing to the observed attenuation of ultrasonic signals. Extensive studies have demonstrated that gas generation—such as CO_2_, CO, C_2_H_4_, and H_2_—is a common result of electrolyte decomposition under various stress conditions, including high voltage, elevated temperature, moisture exposure, and the presence of catalytically active surfaces. The accumulation of these gaseous byproducts within the cell can lead to the formation and expansion of voids or air gaps, which significantly hinder ultrasonic wave propagation. Due to their inherently low acoustic impedance, gases are known to cause substantial ultrasonic signal attenuation. Although other aging mechanisms, such as LAM and continuous growth of the SEI, also contribute to capacity fade, their influence on ultrasonic attenuation appears secondary. In comparison, the physical presence of gas pockets presents a more direct and pronounced barrier to efficient acoustic transmission.

Therefore, for this type of battery, this study can quickly identify batteries with a capacity of less than 240 Ah, significantly improving sorting efficiency. Additionally, once the mapping relationship for a specific battery system is established to determine the threshold value, this method can be extended to the rapid sorting of other types of retired batteries.

## 5. Conclusions

To address the need for rapid and non-destructive detection in the echelon utilization of retired batteries, this paper designs and optimizes an EMAT detection system based on a Halbach magnetic array. Through finite element simulation, the permanent magnet structure and butterfly coil parameters are collaboratively optimized, significantly enhancing the uniformity of the magnetic field and eddy current density. This advancement overcomes the technical limitations of poor penetration associated with traditional ultrasonic coupling agents and air-coupled ultrasonics. Following optimization, the transducer’s SNR is substantially improved, with the amplitude of the received signal increasing by a factor of 4.

Based on the detection system, point scanning tests are conducted on 48 retired 270 Ah batteries. It is found that the critical threshold is 240 Ah capacity—batteries below this value exhibit a 44% attenuation in the amplitude of the ultrasonic transmission signal due to electrolyte drying or gas production. The average amplitude for the 230–240 Ah group is 1.06 V, while the 240 Ah and above group have an average amplitude greater than 1.91 V.

The EMAT system developed in this paper is compatible with thicker batteries, offering enhanced penetration and high environmental adaptability. This system effectively advances battery health assessment from destructive sampling to in situ, non-destructive online detection. It is expected to be applied in the safe reuse of retired batteries in base stations or energy storage scenarios, thereby reducing the overall cost of battery echelon utilization.

## Figures and Tables

**Table 1:** Acoustic parameters of common substances in batteries.

Material	Acoustic Velocity c (km/s)	Density ρ (g/cm^3^)	Acoustic Impedance z (kg/(m^2^·s))
Air	0.344	0.0013	0.00044
Water	1.53	1.0	1.53
LiCoO_2_	6.96	4.92	34.24
LiFeO_4_	7.36	2.88	21.20
Graphite	1.47	2.3	3.38
Aluminum Foil	6.3	2.7	17.01
Copper Foil	4.4	8.9	39.16

**Table 2:** The specific parameters of the transducer.

Parameter	Value	Parameter	Value
Total height of permanent magnet H (mm)	20	The coil wire diameter r (mm)	0.11
Permanent magnet L × D (mm)	20 × 25	Number of turns of the coil N	25
Excitation current frequency f (kHz)	400	The coil line spacing d (mm)	0.08
Excitation current cycle	5	Lift-off distance h (mm)	1

**Table 3:** Summary of ultrasonic testing results for retired batteries grouped by capacity.

Batch Capacity (Ah)	The Number of Batteries	Average Peak Voltage of the Batch (V)	Standard Deviation
230–240	8	1.061	0.621
240–250	17	2.139	0.860
250–260	15	1.906	0.787
260–270	8	1.950	1.235

## Data Availability

The data presented in this study are available on request from the corresponding author.

## Abbreviations

The following abbreviations are used in this manuscript:

EMAT	Electromagnetic acoustic transducer
LIB	Lithium-Ion Battery
PUT	Piezoelectric ultrasonic transducer
SOC	State of charge
ACUT	Air-coupled ultrasonic transducer
LDV	Laser Doppler vibration
SNR	Signal-to-Noise Ratio
